# Phthisis Simulated by Oidium Lesions in Ceylon

**Published:** 1911-12-30

**Authors:** 


### Phthisis Simulated by Oidium Lesions in Ceylon.

The following are three cases illustrative of a malady which occurs in Ceylon, mistakable for phthisis unless care is taken in investigating the nature of the disease, but due, according to Castellani, to an oidium and not to tubercle bacilli.

Mrs. N., a European lady who lived in a very hot and damp district of Ceylon during the previous four years, came under observation complaining that for three months she had been suffering from cough with scanty muco-purulent expectoration, mostly in the morning. Her general condition was fairly good. Examination of the chest revealed no abnormal physical signs. Examination of the sputum for tubercle bacilli gave entirely negative results, but an oidium-like fungus was present in abundance; it was easily cultivated on sugar agars. Sajodin was prescribed in 10-grain doses three times a day, and Dr. Castellani heard later that the patient was much better and that the expectoration had entirely stopped. This was a mild type of the disease; a severer type more closely resembles phthisis. The patient emaciates, there is hectic fever; muco-purulent and bloody expectorations occur, and occasionally true hæmoptysis. Search for tubercle bacilli in the sputum is always negative, as is also the result of guinea-pig inoculations. Physical examination of the chest shows patches of impairment of note, fine crepitations and pleural rubbing. Treatment is of little avail and many of the patients die speedily.

Here are two more of Dr. Castellani's cases:—

Mr. M., a planter, had had several months' illness by the time he reached Colombo; he was then extremely weak, had serotine fever and bloody expectoration. On examination of the chest both sides were found to exhibit several patches of diminished resonance with fine crepitations and pleural rubbings. The sputum was examined for tubercle bacilli many times without any being found, and inoculations into several guinea-pigs all proved negative. Cutaneous and ocular tuberculin reactions were negative. Cultivations were made from the expectoration on several occasions and an oidium-like fungus and a streptococcus were constantly grown. The patient died six weeks later, but no autopsy was allowed.

S. A., a Cingalese man aged twenty-nine years, was admitted to the hospital with a history of continuous fever and cough which were said to be of two weeks' duration. The general condition was fairly good, but the temperature was 101° F. and the pulse-rate 96 per minute. Coughing bouts were frequent, but the muco-purulent expectoration was not abundant. Physical examination of the chest revealed a few rhonchi and some coarse moist râles on both sides; at the right base there was a small patch of impaired resonance and some subcrepitant râles, whilst another patch with similar râles was found in the left infrascapular region. The digestive and circulatory systems seemed natural. There was no thrush of the oral mucosa. The sputum was several times examined for tubercle bacilli, always with negative result; the microscope showed numerous spores and some mycelium of the oidium-like fungus in fresh preparations; it was grown upon maltose and other sugar-agars. Pneumococci were absent. The patient's blood gave a negative Widal reaction, but the sporo-agglutination test with the oidium fungus was positive. The temperature gradually fell to normal. There were no sweatings and no rigors. The condition of the lungs gradually improved, the expectoration, which had never been abundant, decreased and the fungus disappeared.

Dr. Castellani gives the detailed cultural characteristics of this new fungus which he styles *Oidium tropicale*. His general conclusions are as follows:—

1. A type of bronchitis in which an oidium is present in the expectoration is of common occurrence in Ceylon.

2. The fungus is probably the true cause of the disease, for not only could no other causal agent such as tubercle bacilli be found, but also the fungus becomes scarcer and finally disappears.

3. The oidium apparently attacks the bronchi directly, there being no thrush-like affection of mouth or pharynx at the same time.

4. The oidium has distinctive cultural characters and seems to be a new species—*Oidium tropicale*. The pathological condition it causes might be called "tropical broncho-oidiosis."

5. The diagnosis of tropical broncho-oidiosis is based on bacteriological methods, the condition being distinguished from phthisis by the absence of tubercle bacilli, by the negative animal inoculations, and by the negative result of the cutaneous and ocular tuberculin reactions; from broncho-spirochætosis by the absence of spirochætes; and from endemic hæmoptysis by the absence of the ova of the specific worm *Paragonimus Westermani*.

6. Before a diagnosis of broncho-oidiosis is made care should be taken to ascertain that the expectoration was collected in sterile Petri dishes because in a tropical country sputum exposed to the air quickly becomes contaminated with various fungi. Primary broncho-oidiosis should also be distinguished from the secondary broncho-oidiosis occasionally met with in cachectic patients suffering from cancer, diabetes, tuberculosis and so forth, and presenting thrush lesions on the oral mucosa.

